# ﻿A new ghost shrimp of the genus *Pugnatrypaea* (Decapoda, Axiidea, Callianassidae) from the South China Sea

**DOI:** 10.3897/zookeys.1085.79278

**Published:** 2022-02-14

**Authors:** Wenliang Liu

**Affiliations:** 1 Shanghai Key Lab for Urban Ecological Processes and Eco-Restoration, School of Ecological and Environmental Science, East China Normal University, Shanghai, 200241, China East China Normal University Shanghai China; 2 Institute of Eco-Chongming, Shanghai, 200062, China Institute of Eco-Chongming Shanghai China

**Keywords:** Chinese waters, key, *Pugnatrypaearuiyui* sp. nov.

## Abstract

A new species of the genus *Pugnatrypaea* Poore, Dworschak, Robles, Mantelatto & Felder, 2019, *Pugnatrypaearuiyui***sp. nov.**, collected from the northern South China Sea, is described and illustrated. It is distinguishable from *P.pugnatrix* (de Man, 1905), *P.iranica* (Sepahvand, Momtazi & Tudge, 2015), and *P.emanata* Felder & Robles, 2020 in having the lower margin of the male major cheliped merus lacking a proximal hook. It is distinguishable from *P.intermedia* (de Man, 1905) and *P.lobetobensis* (de Man, 1905) in having the upper margins of both cheliped meri unarmed. A key to all species of *Pugnatrypaea* is provided.

## ﻿Introduction

While working on a taxonomic study of the axiidean fauna (Crustacea, Decapoda) of the China seas, an undescribed species assignable to the genus *Pugnatrypaea* Poore, Dworschak, Robles, Mantelatto & Felder, 2019 was found in the northern South China Sea. Poore et al. (2019) established the genus *Pugnatrypaea* on the basis of molecular phylogenetic analyses (Robles et al. 2020) including *P.emanata* Felder & Robles, 2020 (therein = “*Pugnatrypaea* GMX”) and *P.pugnatrix* (de Man, 1905), which together formed a clade; another five species were included in the genus. All seven species share the diagnostic characters including the shape of the third maxilliped merus, third pereopod propodus, uropodal exopod, and telson. Most strikingly, the telson tapers over its distal one-third and ends in a pair of posterior lobes separated by a notch with a medial spine.

Six species are known from the Indo-West Pacific: *P.bicauda* (Sakai, 2010), *P.intermedia* (de Man, 1905), *P.iranica* (Sepahvand, Momtazi & Tudge, 2015), *P.lobetobensis* (de Man, 1905), *P.orientalis* (Bate, 1888), and *P.pugnatrix*. One species, *P.emanata*, is from the Gulf of Mexico (Atlantic). All species occur only in relatively deep continental shelf habitats (Felder and Robles 2020).

## ﻿Materials and methods

All specimens examined have been deposited in the Institute of Oceanology, Chinese Academy of Sciences, Qingdao, China (**IOCAS**). The drawings were made with the aid of drawing tube mounted on a Zeiss Stemi Sv11 compound microscope. Carapace length (cl.) was measured from the tip of the rostrum to the posterior margin of the carapace.

## ﻿Taxonomy

### ﻿Family Callianassidae Dana, 1852

**Genus *Pugnatrypaea* Poore, Dworschak, Robles, Mantelatto & Felder, 2019**

#### ﻿ Pugnatrypaea ruiyui sp. nov.

Taxon classificationAnimaliaDecapodaCallianassidae

87971709-1F97-5313-97A4-212D3C06E25E

http://zoobank.org/F1116F5A-BA90-4D66-B83C-8C44C9B2275C

Figs 1 , 2 , 3 , 4

##### Material examined.

***Holotype***: adult male (cl. 6.8 mm), Q284A-9/MBM210365, Beibu Gulf Stn. 6216, 21°12'N, 108°30'E, 57.5 m, muddy sand, coll. Zhang, 8 November 1960. Paratypes: adult male (cl. 5.9 mm), Q146A-10/MBM210232, Beibu Gulf Stn. 6236, 21°30'N, 108°00'E, 41 m, muddy sand, coll. Fuzeng Sun, 14 February 1960; female (cl. 7.9 mm), Q228A-13/MBM210345, Beibu Gulf Stn. 6217, 21°00'N, 108°30'E, 62.8 m, muddy sand, coll. Jinxi Guan, 11 July 1960; female (cl. 7.2 mm), Q172A-12/MBM210364, Beibu Gulf Stn. 6238, 20°00'N, 108°00'E, 60 m, muddy sand, coll. Jinxi Guan, 13 April 1960; female (cl. 7.9 mm), Q247A-11/MBM210374, Beibu Gulf Stn. 6273, 18°15'N, 106°30'E, 40.3 m, muddy sand, coll. Ruan & Zheng, 14 July 1960.

##### Diagnosis.

Carapace with narrow triangular spiniform rostrum, reaching at least proximal 2/3 of eyestalk in dorsal view; dorsal oval well defined. Eyestalk elongate, subrectangular, distomedial corner produced into rounded prominence, cornea well defined. Antennular peduncle much shorter than antennal peduncle. Third maxilliped lacking exopod, row of spines forming distinct crista dentata on internal surface of ischium. Male major cheliped merus lacking proximal hook on lower margin, instead with nine minute spines on middle part. Pleomeres smooth dorsally, dorsal tergite fused with the lateral pleuron, pleomere 2 distinctly longer than other pleomeres. Pleopod 1 uniramous, pleopod 2 biramous in both males and females. Third through fifth pleopodal endopods each with slender, finger-like appendix interna extending clearly beyond margin. Telson elongate, subrectangular, posterior margin distinctly bilobate, lobes posteriorly separated by deep incision accommodating distinct median spine. Uropodal endopod broad, ~1.1× longer than broad, with spiniform setae near anterior and distal margins and a movable spine near posterolateral angle; exopod ~1.2× as long as wide, anterodistal corner right-angled, posterodistal margin with row of 6–8 long, blade-like setae proximal to long setae on distal margin.

##### Description.

**Figure 1.**
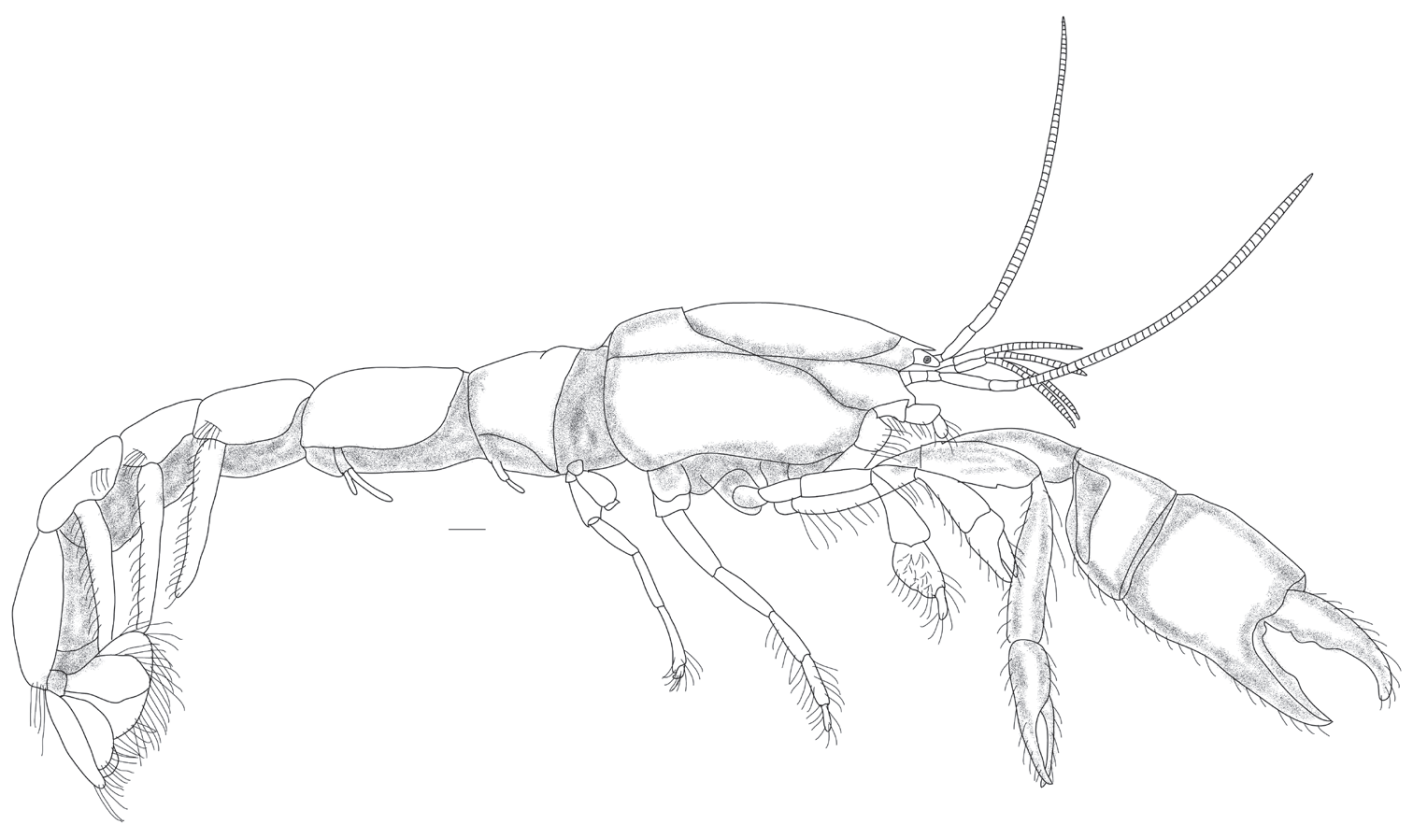
*Pugnatrypaearuiyui* sp. nov., holotype male (Q284A-9/MBM210365, CL 6.8 mm), entire animal, lateral view. Scale bar: 1 mm.

**Figure 2.**
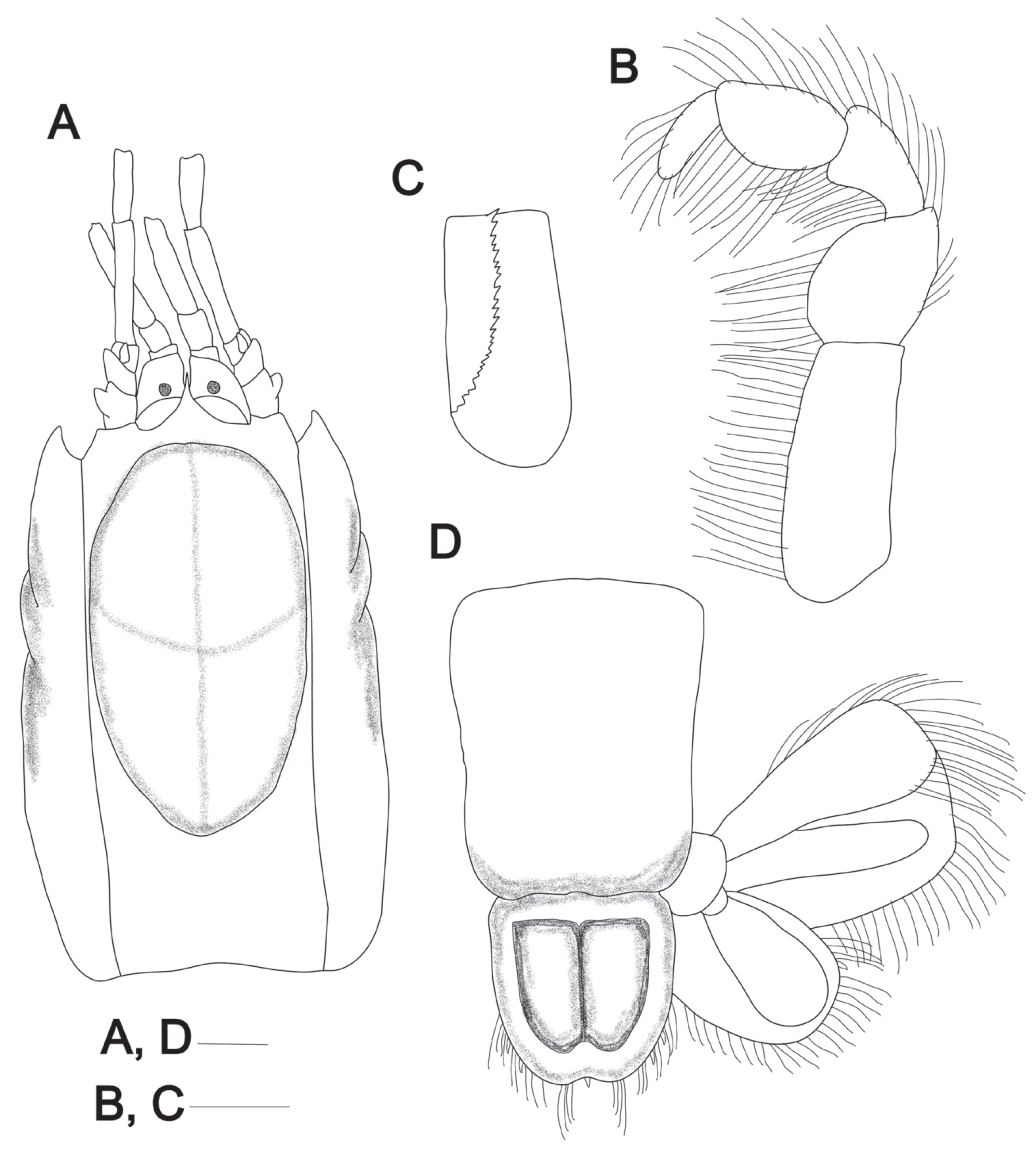
*Pugnatrypaearuiyui* sp. nov. **A, D** holotype male (Q284A-9/MBM210365, CL 6.8 mm) **B, C** paratype female (Q228A-13/MBM210345, CL 7.9 mm) **A** carapace, dorsal view **B** left maxilliped 3, outer view **C** ischium of left maxilliped 3, inner view **D** telson and uropods, dorsal view. Scale bar: 1 mm.

Rostrum (Figs 1, 2A) acute, narrowly triangular, flexure weakly sinuous in lateral view, terminally spiniform, tip slightly upturned, reaching at least 2/3 of proximal eyestalk in dorsal view. Carapace smooth (Figs 1, 2A), ~0.3 of total body length; dorsal oval well defined, 0.6× as long as carapace. Cervical groove located at posterior quarter; linea thalassinica complete.

Eyestalks (Fig. 2A) elongate, subrectangular in dorsal view, lateral margin distinctly sinuous, reaching 4/5 length of first article of antennular peduncle; cornea subterminal, disk-shaped, with scattered, brown spots, corneal width less than 1/3 of basal width of eyestalk.

Antennular peduncle (Fig. 2A) shorter and not strikingly heavier than antennal peduncle, extending beyond proximal end of distal article of antennal peduncle; second article shorter than basal, third article ~2.5× length of second; outer flagellum and inner flagellum shorter than peduncle. Antennal peduncle (Fig. 2A) reaching approximately to mid-length of antennular flagellum rami; basal article slightly longer than second article; length of second article ~2× width, distal articulation to third article overreached dorsally by rudimentary spiniform scaphocerite; fifth article distinctly shorter than fourth article.

Third maxilliped (Fig. 2B, C) pediform, lacking exopod; endopod fringed by long setae; ischium subrectangular, length ~1.75× breadth, with slightly angled longitudinal row of spiniform teeth forming strong crista dentata on internal surface; merus subovate, slightly longer than wide, ~2/3 length of ischium; carpus almost as broad as propodus, both longer than wide; propodus ovate; dactylus fingerlike, weakly arcuate, 3.3× as long as wide, 0.75× length of propodus.

**Figure 3.**
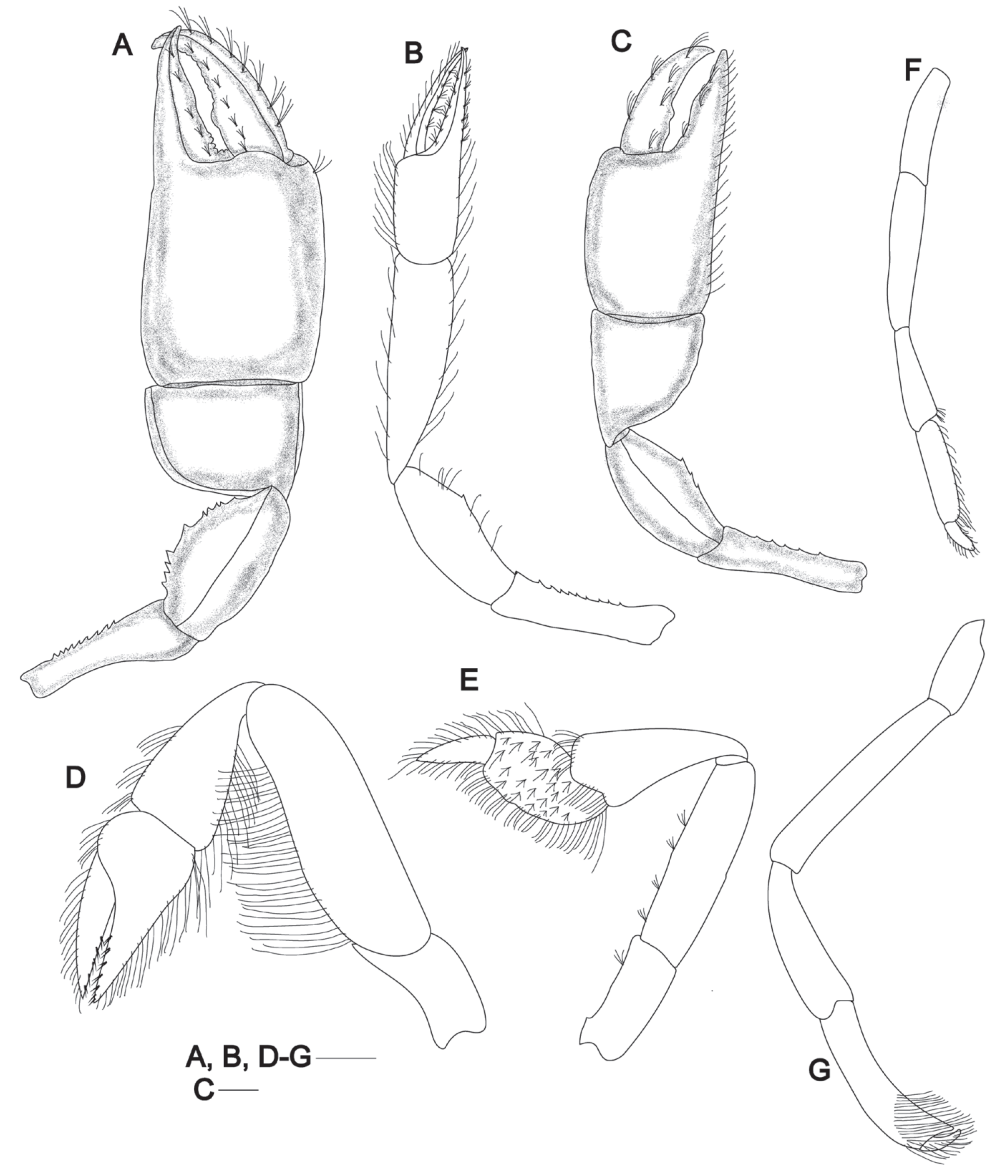
*Pugnatrypaearuiyui* sp. nov. **A, B** paratype male (Q146A-10/MBM210232, cl, 5.9 mm) **C** paratype female (Q247A-11/MBM210374C, cl, 7.9 mm) **D–G** paratype female (Q228A-13/MBM210345, cl, 7.9 mm) **A** male left larger cheliped, outer view **B** male right smaller cheliped, outer view **C** female right larger cheliped, outer view **D–G** pereopods 2–5, outer view. Scale bar: 1 mm.

Pereopods 1 unequal and strongly dissimilar, major cheliped located on either right or left side, shape, and ornamentation sexually dimorphic. Male major cheliped massive (Fig. 3A). Ischium 2.8× as long as high; upper margin sinuous, unarmed; lower margin slightly convex, armed with row of nine inconspicuous subequal spines. Merus as long as ischium, 1.8× as long as high; upper margin slightly convex, unarmed; lower margin sinuous, with row of nine minute spines on middle part, the middle one largest. Carpus elongated, 0.7× as long as high, 0.7× as long as merus; upper margin almost straight; proximo-lower margin gently convex, unarmed. Chela ~2.0× as long as high; palm subquadrate, ~1.3× as long as high, 1.9× as long as carpus; fixed finger 0.6× as long as palm, cutting edge with some inconspicuous denticles basally; dactylus slightly curved distally, slightly longer than fixed finger, cutting edge sinuous, armed with row of inconspicuous denticles and a large round denticle in the middle.

Male minor cheliped (Fig. 3B) shorter and much more slender than larger cheliped. Ischium ~4.0× as long as high, upper margin nearly straight, unarmed, lower margin with row of nine inconspicuous subequal spines. Merus 0.9× as long as ischium, ~2.4× as long as high, upper and lower margins slightly convex, lower margin with a small upper-middle spine; outer surface medially swollen. Carpus 3.5× as long as high, 1.4× as long as merus, abruptly narrowed at base; upper margin almost straight; proximo-lower margin convex. Chela with narrow gap between slender dactylus and fixed finger; palm nearly 1.5× as long as high, 0.5× as long as carpus; fixed finger tapering distally to acute tip, cutting edge unarmed; dactylus slightly longer as palm, slender, unarmed on concave cutting edge.

Female major cheliped (Fig. 3C) with ischium 3.5× as long as high, upper margin slightly sinuous; lower margin almost straight, with row of five inconspicuous subequal spines. Merus ~0.9× as long as ischium, upper margin slightly convex, unarmed; lower margin slightly convex, with three small spines at mid length. Carpus broad, subquadrate, 1.2× as long as high, 0.9× as long as merus; upper margin almost straight, lower margin keeled. Chela similar to that of male but relatively smaller; palm subquadrate, ~1.5× as long as high; fixed finger 0.7× as long as palm, cutting edge with a wide triangular denticle on middle; dactylus slightly longer than fixed finger, cutting edge sinuate and with two round denticles on proximal half. Minor cheliped in female similar to that of male and ~0.8× as long as major cheliped.

Pereopod 2 (Fig. 3D) chelate. Ischium 1.8× as long as high; merus ~3.3× as long as high, upper margin smooth, lower margin protruding and with row of dense long setae; carpus subtriangular, shorter than merus; chela slightly shorter than carpus, with dense setae on lower and upper margins; palm with upper margin slightly convex; dactylus 2.3× as long as upper margin of palm; carpus and chela fringed with short to long setae along margins.

Pereopod 3 (Fig. 3E) simple, moderately slender. Ischium slender, ~2.0× as long as high; merus ~3.7× as long as high; carpus subtriangular, shorter and broader than merus, broadest subdistally, ~2.4× as long as high; propodus subrectangular, lower margin round, upper margin slightly convex and 0.4 length of carpus, with numerous tufts of setae on lateral surface and row of thick setae along upper and lower margin, no distinct heel delimited; dactylus subtriangular, upper and lower margin slightly convex, outer surface densely setose, terminating in corneous tip.

Pereopod 4 (Fig. 3F) slender, semichelate, all articles unarmed. Ischium rectangular; merus ~1.4× as long as ischium; carpus 0.7 length of merus; propodus 0.9 length of carpus, lower margin densely setose; dactylus elongate, tapering distally, setose on lateral margin.

Pereopod 5 (Fig. 3G) slender, semichelate, all articles unarmed. Ischium rectangular; merus ~2.3× as long as ischium; carpus ~0.7 length of merus, upper margin swollen; propodus almost as long as carpus, lower distal corner projecting to form a chela with dactylus, lateral surface beset distally with dense setae; dactylus hooked toward external side of fixed finger, tips of dactylus and fixed finger obtuse.

Pleomeres smooth dorsally. Pleomere 1 narrowing anteriorly in dorsal view; dorsal tergite fused with the lateral pleuron; pleuron weakly developed but with clearly defined ventral margin. Pleomere 2 distinctly longer than other pleomeres, with posterolateral margin of pleuron slightly expanded. Pleomeres 3–5 with pleura each having tuft of moderately long plumose setae. Pleomere 6 (Fig. 2D) rectangular in dorsal view, very slightly narrowed posteriorly; lateral margin smooth, with inconspicuous notch.

**Figure 4.**
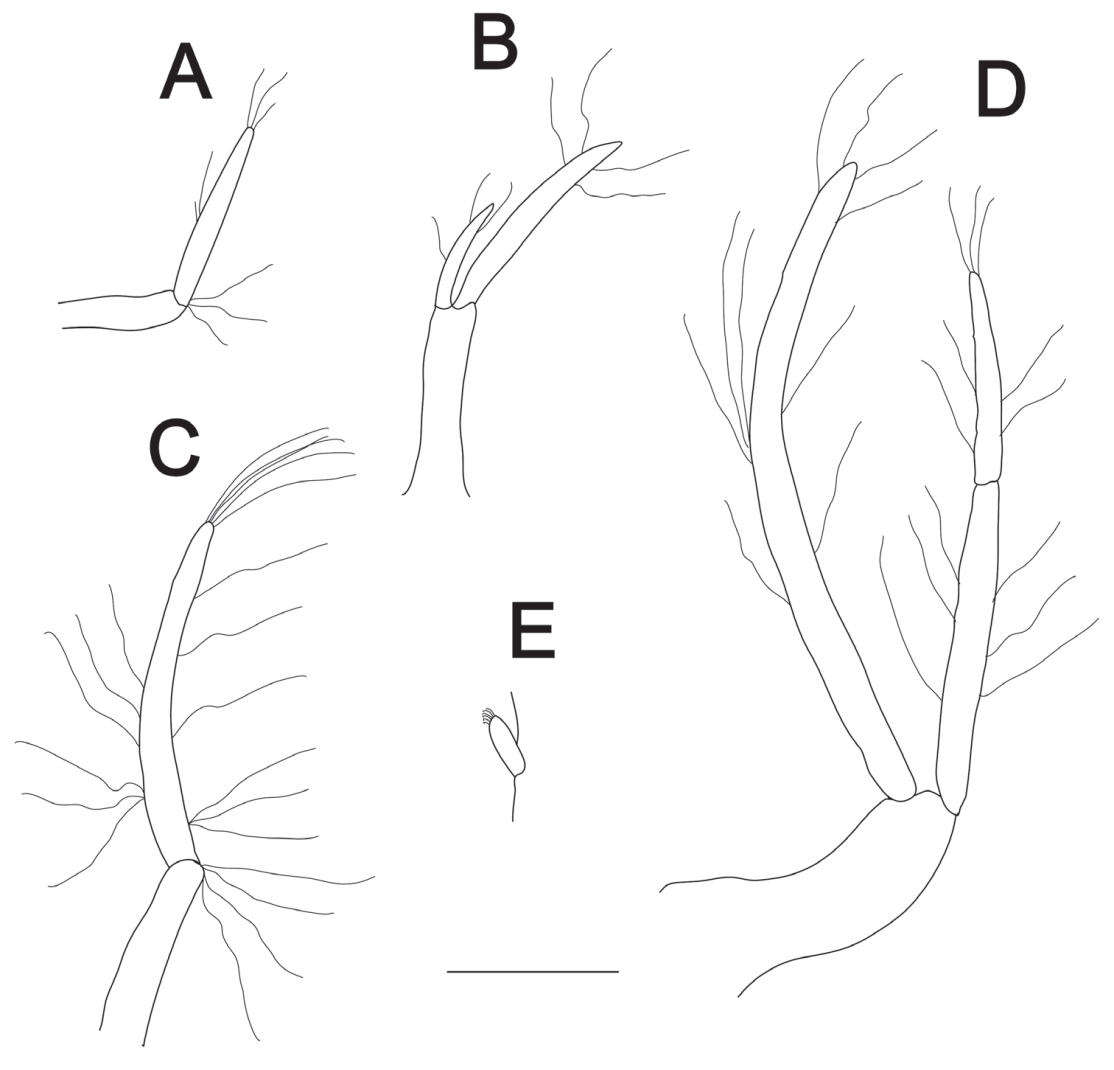
*Pugnatrypaearuiyui* sp. nov. **A, B** holotype male (Q284A-9/MBM210365, cl, 6.8 mm) **C–E** paratype female (Q228A-13/MBM210345, cl, 7.9 mm) **A** male pleopod 1, posterior view **B** male pleopod 2, posterior view **C** female pleopod 1, posterior view **D** female pleopod 2, posterior view **E** appendix internae of pleopod 3, posterior view. Scale bar: 1 mm.

Male pleopod 1 (Fig. 4A) uniramous, biarticulated; distal article with some distal setae. Male pleopod 2 (Fig. 4B) biramous, exopod uniarticulated, ~ 2.0 × as long as endopod, sinuous, bearing some long setae distally; endopod with short setae terminally. Female pleopod 1 (Fig. 4C) uniramous, biarticulated; protopod article sinuous, shorter than ramus; ramus spatulate distally and weakly thickened basally, bearing long setae on both margins. Female pleopod 2 (Fig. 4D) biramous; exopod biarticulated, shorter than endopod, sinuous, bearing some long setae on both margin and distally; endopod with long setae on middle part and short setae terminally. Pleopods 3–5 biramous, foliaceous, rami broad; appendix internae (Fig. 4E) slender, finger-like, distinctly projecting beyond margin of endopod, bearing numerous small adhesive hooks along mesial margin.

Telson (Fig. 2D) elongate subquadrate, almost as long as wide and 0.6× as long as pleomere 6, lateral margins with weakly projecting lateral lobes in anterior 1/4, margins weakly converging posteriorly, lateral margins each with low convexity proximally and two pairs of spiniform setae near posterolateral angle; posterior margin deeply concave and bilobed, with distinct median spine and long spiniform setae; dorsal surface with weak median elevation in anterior 1/3.

Uropodal endopod (Fig. 2D) ovate, shorter than telson, ~1.1× as long as wide; anterior margin slightly convex, posterodistal margin evenly convex, with spiniform setae near anterior and distal margins and a movable spine near posterolateral angle; with distinct submedian carina on dorsal surface. Uropodal exopod (Fig. 2D) subrectangular, ~1.2× as long as wide; distal margin clearly differentiated from anterior margin, anterodistal corner right-angled, posterodistal margin with row of 6–8 long, blade-like setae proximal to long setae on distal margin; with a distinct submedian carina on dorsal surface.

##### Remarks.

*Pugnatrypaearuiyui* sp. nov. is the eighth species assigned to the genus *Pugnatrypaea* on account of the spiniform rostrum; telson anterolateral lobe obsolete, undefined, tapering over distal 1/3 ending in pair of posterior lobes separated by notch, with medial spine.

The morphological separation of *Pugnatrypaearuiyui* sp. nov. from its congeners draws upon the work of Bate (1888), de Man (1928a, 1928b), Sakai (1999; 2010; 2011), Sepahvand et al. (2015), and Felder and Robles (2020). The new species is closely related to *P.intermedia* and *P.lobetobensis* in lacking a proximal hook on the lower margin of the male major cheliped merus, whereas *P.pugnatrix*, *P.iranica*, and *P.emanata* bear a proximal hook, but it can be distinguished from latter two in having the upper margins of the meri of both chelipeds meri (versus with proximal spines). Unfortunately, both *P.bicauda* and *P.orientalis* are known from only single, apparently incomplete specimens that lack both chelipeds. The new species can be distinguished from *P.bicauda* by the biramous male pleopod 2 (versus uniramous) and differs markedly from *P.orientalis* in having the rostrum shorter than the eyestalks (versus rostrum slightly extending beyond the tip of the eyestalks).

##### Etymology.

The species is named in honor of the late Professor Ruiyu Liu (J.Y. Liu), of the Institute of Oceanology, Chinese Academy of Sciences, for his great contributions to Chinese carcinology.

##### Distribution and habitat.

Presently only known from the type locality, at depths of 40–63 m on muddy sand substrates.

### ﻿Key to the species of the genus *Pugnatrypaea*

**Table:** 

1	Eyestalk with a sharp distal spine	***P.iranica* (Sepahvand, Momtazi & Tudge, 2015)**
–	Eyestalk unarmed	**2**
2	Rostrum slightly extending beyond tip of eyestalks	**3**
–	Rostrum shorter than eyestalks	**4**
3	Pleomere 6 1.4× as long as telson	***P.orientalis* (Bate, 1888)**
–	Pleomere 6 1.2× as long as telson	***P.intermedia* (de Man, 1905)**
4	Telson 1.3× as long as wide	***P.bicauda* (Sakai, 2010)**
–	Telson almost as long as wide or length slightly exceeding width	**5**
5	Upper margins of merus in both chelipeds with proximal spines	***P.lobetobensis* (de Man, 1905)**
–	Upper margins of merus in both chelipeds unarmed	**6**
6	Male major cheliped merus without proximal hook on lower margin, but with 9 minute spines on middle part	***P.ruiyui* sp. nov.**
–	Male major cheliped merus with proximal hook on lower margin	**7**
7	Proximal hook on lower margin of male major cheliped merus broader, terminating in single acute tip or with ancillary subterminal spine or small denticles	***P.emanata* Felder & Robles, 2020**
–	Proximal hook on lower margin of male major cheliped merus slender and sharply, terminating in single acute tip	***P.pugnatrix* (de Man, 1905)**

## Supplementary Material

XML Treatment for Pugnatrypaea ruiyui
